# Isothermal Crystallization Kinetics Study of Fully Aliphatic PA6 Copolyamides: Effect of Novel Long-Chain Polyamide Salt as a Comonomer

**DOI:** 10.3390/polym11030472

**Published:** 2019-03-12

**Authors:** Syang-Peng Rwei, Palraj Ranganathan, Yi-Huan Lee

**Affiliations:** 1 Institute of Organic and Polymeric Materials, National Taipei University of Technology, Taipei 10608, Taiwan; rangapalraj@gmail.com (P.R.); yihuanlee@mail.ntut.edu.tw (Y.-H.L.); 2 Research and Development Center for Smart Textile Technology, National Taipei University of Technology, Taipei 10608, Taiwan

**Keywords:** BABA/SA salt, PA6-BABA/SA, isothermal crystallization kinetics, nucleating seeds

## Abstract

N^1^, N^6^-bis (4-aminobutyl) adipamide (BABA) diamine and sebacic acid (SA), also called BABA/SA polyamide salt, were used in a typical melt polymerization processes of polyamide 6 (PA6) to form a series of PA6-BABA/SA copolyamides. The effects of BABA/SA on the isothermal crystallization kinetics of PA6-BABA/SA were studied for the first time. An isothermal crystallization analysis demonstrates that the PA6-BABA/SA matrix provided a higher crystallization rate and shorter half-crystallization time than virgin PA6 did. The degree of crystallization of the PA6-BABA/SA_30_ matrix was also the lowest among all of the samples considered herein. This result is attributed to the high nucleation efficacy of a small amount of BABA/SA in the crystallization of PA6. Values of the Avrami exponent (*n*) from 1.84 to 3.91 were observed for all of the polyamide samples, suggesting that the crystallization was involved via a two- to three-dimensional growth mechanism. These findings deepen our understanding of the structure–property relationship of PA6-BABA/SA copolyamides, favoring their practical application.

## 1. Introduction

Nylons, also known as polyamides, are important commercial engineering thermoplastic materials that are used in a large range of applications owing to their excellent properties, including their high temperature- and corrosion-resistance, toughness, and modulus, as well as their anti-fatigue and oil-proof characteristics [1,2]. Of these polyamides, polyamide 6 (PA6) is the most representative and most common. PA6 is the most frequently used and most useful semi-crystalline engineering thermoplastic polyamide, owing to its superior mechanical strength, high stiffness, and chemical resistance against a wide range of solvents and hydrocarbons [3,4]. Accordingly, its mechanical properties and crystal structure have been studied intensively [5,6]. PA6 crystallizes into two well-known crystal structures—α-type and γ-type polymorphs [7,8]. The monoclinic α-crystalline phase of PA6 is the most commonly observed. In its thermodynamically stable phase, the chain is in the fully extended configuration in an anti-parallel fashion, whereas in the thermally less stable pseudohexagonal γ-phase, the parallel chains are twisted approximately 60° out of the plane of pleated (molecular) sheets [9,10]. The crystallization mechanism of the semi-crystalline PA6 has been extensively investigated during the recent decades, and its effects on the final properties of the polymer materials remain the subject of numerous scientific studies [11,12]. The rapid crystallization of PA6 can cause some difficulties during processing in the melt state. A high degree of mold shrinkage and dimensional instability may occur, detrimentally affecting the ultimate properties of this polymer material [13]. Therefore, the degree of crystallinity and the parameters of crystallization are valuable information for the development of products using PA6.

In recent years, PA6-based copolymers have been widely studied from both scientific and technological perspectives, owing to their remarkable properties. Inserting co-constituents (such as comonomers, stereoisomers, branches, and organic and/or inorganic fillers) can yield PA6 copolymers with interesting physical and mechanical properties [14]. The crystallization of the PA6 copolymer is strongly affected by the presence of a second component. The properties of the PA6 copolymers depend markedly upon the structure and amount of co-monomers, as well as the crystallization conditions. Several investigations have established that comonomers can act as nucleating seeds, increasing the rate and degree of crystallization. For example, Wang et al. [15] examined the isothermal crystallization kinetics of PA6/polyethylene-octene (POE) blends, and found that POE had a heterogeneous nucleating effect on the PA6 matrix. Murthy et al. [16] revealed that adding polyamide 66 (PA66) and amorphous nylon (PA6I/T) comonomer units into the PA6 backbone reduced the rate of crystallization. Zhou et al. [17] and Gogolewski et al. [18] obtained similar results.

PA6 copolymers that incorporate bio-based monomers have attracted the attention of numerous researchers, as they considerably reduce the carbon footprint of, and have a positive effect on the value over the life cycle of, plastic products. Substantial effort is being made in the research into the potential of bio-based monomer-derived PA6 copolyamides. In some studies, a series of PA6 copolyamides have been synthesized [19,20]; however, a detailed investigation on the effect of the amount of the second component on a copolymer of PA6 and a bio-based monomer, as well as on its isothermal crystallization kinetics, is lacking. The isothermal crystallization process is very amenable to theoretical analysis. Very few studies have addressed the effect of a bio-based monomer or bio-based polymer derivatives on the crystallization properties of PA6. In an earlier work [21], we synthesized PA6/6T and PA66/6T, and studied the crystallization kinetics and morphologies of these novel copolyamides. We found that the crystallization behavior of these two series of copolyamides may be regulated by the types of monomers and their amount. In a subsequent investigation [22], we synthesized a series of PA6 copolyamides (PA6-N^1^, N^6^-bis (4-aminobutyl) adipamide (BABA)/sebacic acid (SA)) using novel long-chain diamine (N^1^, N^6^-bis (4-aminobutyl) adipamide) diamine, BABA), and polyamide salt (BABA/SA) monomers. These polyamides have low-melting points that are comparable to that of neat PA6. Hence, the modification of PA6 using a small amount of polyamide salt as a comonomer can yield PA6-BABA/SA with excellent properties, owing to its long-chain flexible segments. The physical, chemical, and mechanical properties of polyamides are well known to be able to be affected by crystallization kinetics, and these relationships powerfully support the analysis and design of the processing operations. Consequently, isothermal crystallization kinetics were studied using differential scanning calorimetry (DSC).

The current study focuses on the effects of the BABA/SA content on the crystal morphology and isothermal crystallization processes of PA6-BABA/SA copolyamides, which are compared with those of virgin PA6. The crystallization kinetic parameters are estimated, and the relative crystallinities of virgin PA6 and PA6-BABA/SA are examined.

## 2. Experiment

### 2.1. Materials

All of the reagents were used as received; dimethyl adipate (DMA; ≥99%), 1,4-diaminobutane (BDA; ≥99%), sebacic acid (SA; ≥99%), ethanol (≥99.5%), methanol (≥99.8%), diethyl ether (≥99%), toluene (≥99.8%), deuterated deuterium oxide (D_2_O-d; ≥99.9%), sulfuric acid (≥99.99%), deuterated trifluoroacetic acid (TFAA-d; ≥99.5%), ε-caprolactam (CPL; 99%), and 6-aminocaproic acid. 

### 2.2. Method 

**Scheme 1:**
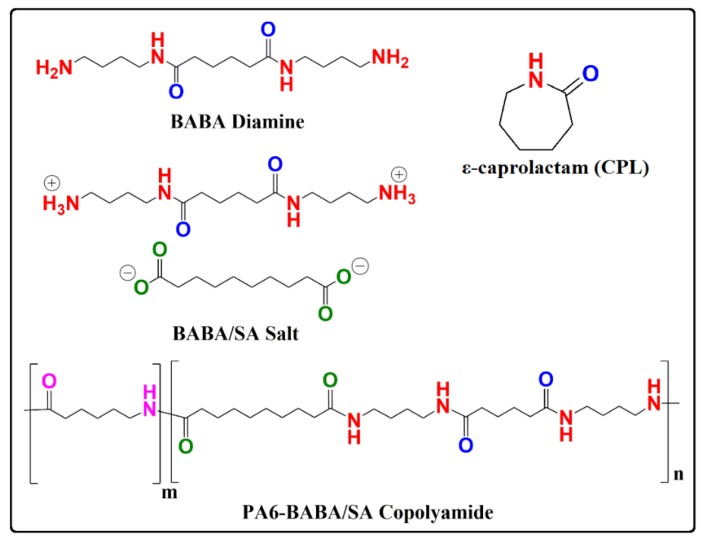
Chemical structures of monomers and polyamide 6-N^1^, N^6^-bis (4-aminobutyl) adipamide-sebacic acid (PA6-BABA/SA) copolyamides.

BABA (N^1^, N^6^-bis (4-aminobutyl) adipamide) diamine, BABA/SA salt, and PA6-BABA/SA copolyamides (Scheme 1) were synthesized in our laboratory, as described in our earlier work [22]. Their chemical structure was confirmed by ^1^H NMR and FTIR spectroscopy. The copolymer composition of PA6 and of the BABA/SA segment are calculated from the signal integrals of peaks at *a*, *b*, *c*, and *f* using the following equations, for which the results are presented in Table 1.

(1) $$a+b=\;\frac{x}{8}+\frac{y}{2}$$

(2) $$c+f=\;\frac{y}{6}+\frac{x}{18}$$

(3) $$PA6=\;\frac{y}{x+y}\;\times \;100\%;\;\;\;\;\frac{BABA}{SA}=\;\frac{x}{x+y}\;\times \;100\%$$

where, *x* and *y* are the mole % of BABA/SA and PA6, respectively. 

### 2.3. Measurements 

#### 2.3.1. Nuclear Magnetic Resonance (NMR) Spectroscopic Analysis 

The room-temperature ^1^H NMR spectra of PA6, and a series of PA6-BABA/SA were recorded in TFAA-d using a Bruker Avance 400 MHz spectrometer (Billerica, MA, USA). The solvent signal was used as a reference.

#### 2.3.2. Fourier Transform Infrared Spectroscopic (FTIR) Analysis 

The FTIR spectra of the polymer samples were obtained using a Nicolet 5700 FTIR spectrometer (Thermo Fisher Scientific, Waltham, MA, USA). The measurements of all of the samples were made with a signal average of 32 co-added scans and a resolution of 4 cm^−1^, within a wave number range of 500–4000 cm^−1^, using a KBr pellet.

#### 2.3.3. Viscometry

Reduced viscosity (RV) measurements were made using 1.0 g/DL of polyamides in formic acid (90 ± 1%) at 25 ± 0.05 °C, using an Ubbelhode Viscometer. The RV of the polyamides was calculated using the solvent flow time and the polymer solution flow time in the viscometer. The molecular weight (*M*_w_) of the PA6-BABA/SA copolyamides were obtained from our previously published literature [22]. 

#### 2.3.4. Differential Scanning Calorimetry (DSC)

A DSC (SHIMADZU AGS-X 500 N, Waltham, MA, USA) was used to determine the melting temperatures (*T*_m_) of the polyamide samples. Samples with masses of 3–5 mg were placed in aluminum crucibles. Each sample was heated from 60–250 °C at a heating rate of 10 °C/min under a nitrogen flow of 50 mL/min, which was maintained for 3 min, before they were cooled to 60 °C and then cooled down to 60 °C at a cooling rate of 10 °C/min. 

#### 2.3.5. Dynamic Mechanical Analysis (DMA)

DMA was used to analyze the glass transition temperature (*T*_g_) of the series of all of the polymer samples (TA instrument, Q 800, Newcastle, DE, USA). Tan δ was obtained by heating each sample (15 mm × 15 mm × 2 mm) from −20 to 125 °C at a heating rate of 10 °C/min. The data were collected at a frequency of 3 Hz in a compression mode.

#### 2.3.6. Isothermal Crystallization Process by DSC

The DSC measurements were made using a SHIMADZU AGS-X 500 N (Waltham, MA, USA), which was calibrated to measure the temperature and heat of fusion using standard indium (*T*_m_ = 156.6 °C and Δ*H* = 28.5 J/g). All of the DSC experiments were conducted under a nitrogen purge at a constant flow rate (20 mL/min) in order to minimize oxidation, and the sample weights were 5–8 mg. Below the *T*_m_ of polymer samples, there is a definite dependence of the nucleation rate on the melt conditions. Therefore, all of the samples were melted above the melting temperatures. Crystallization temperatures of 178–188 °C, 162–166 °C, 132–146 °C, and 127–143 °C were chosen for all of the polymer samples, the range being slightly below the melting point of the polymers, which allowed for carrying out the DSC measurements. At temperatures lower than *T*_c_, the crystallization of all of the polymer samples begins, before they are cooled to the proper temperature. Therefore, the crystallization temperature of the end point of the transition was taken as the transition temperature for the isothermal crystallization study. The isothermal crystallization and melting process were performed as follows: the samples were heated quickly (at 80 °C/min) to 20–30 °C above the melting temperature (*T*_m_), stayed there for 10 min to eliminate residual crystals, then cooled (at –80 °C/min) to the designated crystallization temperatures (*T*_c_) in the range of 178–188 °C, 162–166 °C, 132–146 °C, and 127–143 °C for PA6, PA6-BABA/SA_30_, PA6-BABA/SA_50_, and PA6-BABA/S_70_ for the isothermal crystallization for 10 min, and then heated to 250 °C at a rate of 10 °C/min. The heat of fusion was obtained by integrating the area under the *T*_m_ peaks of the DSC curves, and was directly proportional to the degree of crystallinity. The heat of fusion of the 100% crystalline PA6 was taken as 188 J/g, and was used to determine the weight percentage crystallinities of virgin PA6 and PA6-BABA/SA. The degree of crystallinity (*X*_C%_) of a copolymer was determined using Equation (4).

(4) $$X_{c}\;\left(\%\;\mathrm{crystallinity}\right)=\;\left(\frac{\Delta H_{f}}{\left(1-\Phi \right)\Delta H^{*}}\right)$$

where $\Delta H_{f}$ denotes the heat of fusion of the PA6-BABA/SA copolyamide samples, $\Delta H^{*}$ is the heat of fusion of the 100% crystalline neat PA6, and Φ is the weight fraction of BABA/SA in the PA6 sample. The reported crystallinity values are the means of at least three determinations. 

#### 2.3.7. X-Ray Diffraction (XRD) Analysis

XRD patterns were obtained using a Bruker D8 Discover powder diffractometer (Malvern Ltd, Malvern, UK) with Cu Kα radiation, λ = 0.1542 nm, a voltage of 40 (kV), and a current of 100 mA. The tested specimen samples were scanned from 2θ = 10 to 50° with a scanning rate of 0.2°/min. 

#### 2.3.8. Polarizing Microscopy (POM)

POM observations were made with a XP-203 polarizing microscope (Chang fang Optical Instrument, Shanghai, China) equipped with a hot stage and charge coupled device (CCD) camera. The samples were melted at 250 °C between two glass slides so as to obtain thin films, and were maintained at this temperature for 10 min. The films were transferred onto another hot stage with a pre-set crystallization temperature for 30 min.

## 3. Results and Discussion

### 3.1. Characterization of PA6 and PA6-BABA/SA Copolyamides

The polyamide salt was melt-polymerized with PA6 at weight percentages of 30%, 50%, and 70% of the total weight of the composition. The mixtures thus obtained are denoted as PA6-BABA/SA_30_, PA6-BABA/SA_50_, and PA6-BABA/SA_70_, respectively. Our previous study clearly described the mechanism of the synthesis of PA6-BABA/SA [23]. 

**Figure 1:**
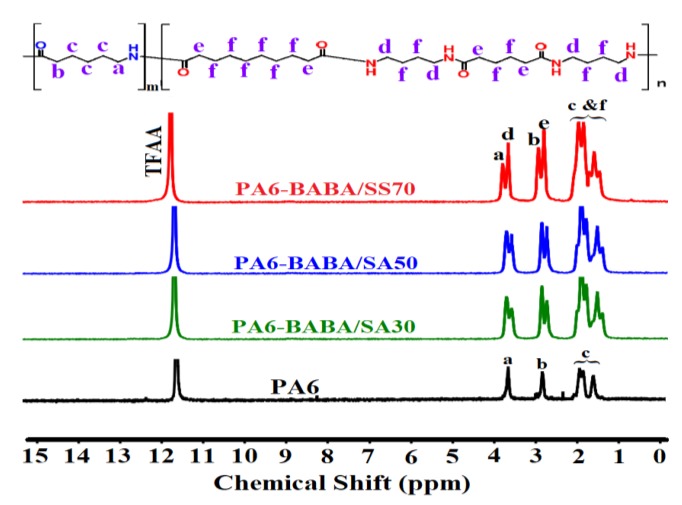
^1^H NMR spectra of virgin PA6 and PA6-BABA/SA with various wt % BABA/SA.

The chemical structures of the as-synthesized copolyamides were characterized by ^1^H NMR and FTIR spectroscopy. Figure 1a shows the ^1^H NMR spectra of virgin PA6. The proton signals in the regions around 3.61 ppm (peak a) and 2.81 ppm (peak b) were attributed to the chemical shift of the methylene protons adjacent to the amino (-CH_2_-NH-CO-) group and carbonyl group (-NH-CO-CH_2_) of PA6, respectively. The proton peaks in the region from 2.08 to 1.30 ppm (peak c) are associated with the other protons in the aliphatic methylene units of PA6. After the incorporation of BABA/SA with PA6 (Figure 1b–d), new signals were detected in the regions around 3.47 ppm (peak d), 2.56 ppm (peak e), and 2.08 to 1.30 (peak c and f), which were attributed to the chemical shift of the methylene protons adjacent to the amino (-CH_2_-NH-CO-) and carbonyl groups (-NH-CO-CH_2_), as well as the other methylene protons of BABA/SA, respectively. Therefore, the peaks (a, b, and c) were assigned to virgin a PA6 unit, and the peaks (d, e, c, and f) were assigned to a BABA/SA unit, indicating the successful synthesis of PA6-BABA/SA copolyamides [22]. 

**Figure 2:**
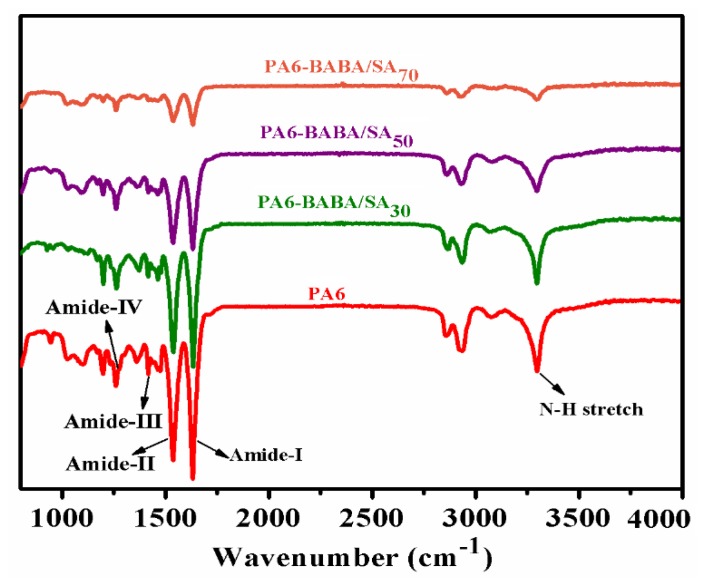
FTIR spectra of virgin PA6 and PA6-BABA/SA with various wt % of BABA/SA.

Figure 2 displays the FTIR spectra of virgin PA6 and a series of PA6-BABA/SA. The absorption band at 3302 cm^−1^ is attributable to the hydrogen-bonded V_N-H_ stretching vibration. The coupled bands at 2857 and 2952 cm^−1^ (symmetric and asymmetric) are ascribed to the stretching vibration of V_C-H_. All of the characteristic peaks of the amide groups are listed as follows: 1635 cm^−1^ (amide I, C=O stretching vibration), 1535 cm^−1^ (amide II, C–N stretching, and CO–N–H bending vibration), 1461 cm^−1^ (amide III, C–N stretching, and C–H in-of-plane bending vibration), 1262 cm^−1^ (amide IV, C–CO stretching vibration), 720 cm^−1^ (CH_2_ wagging), and 686 cm^−1^ (amide V, N–H out-of-plane bending vibration). The FTIR spectra are in correlated with the previously reported data [22], and confirm the anticipated chemical structure of these copolyamides. 

### 3.2. RV and M_*w*_

The *RV* and *M*_w_ of copolyamides (see Table 1) decreased as 30 wt % of BABA/SA was incorporated, reaching their minimum values at a BABA/SA content of 30 wt %. The trend of decrease in the RV and *M*_w_ is in accordance with the reduction in chain stiffness. However, the RV and *M*_W_ of the copolyamides increased smoothly with the increase in the BABA/SA salt (50–70 wt %) content. As a result, the higher concentration of nylon salt (BABA/SA) caused the formation of larger amounts of reaction water, which could promote the hydrolysis of macromolecules and shorten the polyreaction time, as the polymerization rate of BABA/SA is faster than that of CPL. Therefore, the copolymerization rate became greater with the incorporation of an increasing amount of BABA/SA (50–70%), and resulted in the molecular masses of copolyamides, which were reflected by the greater RV. The increasing polymerization rate with the increasing wt % of the BABA/SA content was attributed to the superior polyamidation between PA6 and BABA/SA [22].

### 3.3. Crystal Structure

**Figure 3:**
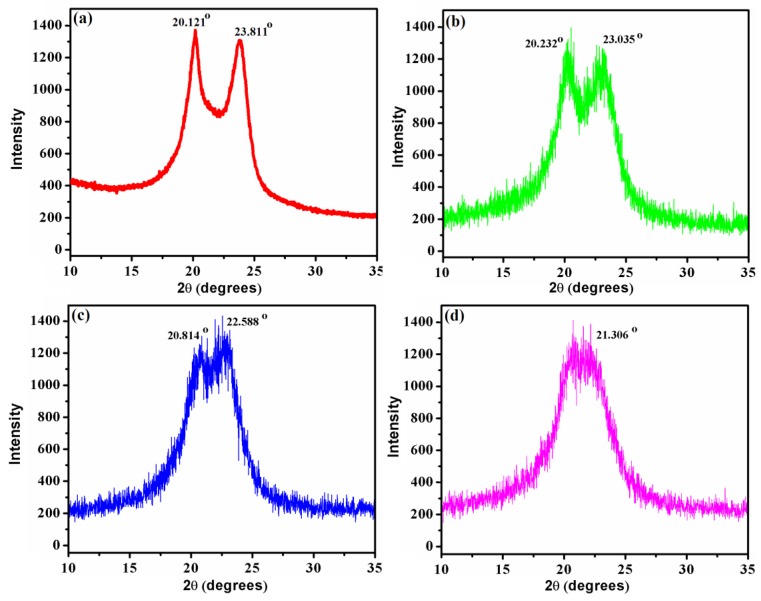
X-ray diffraction pattern of (**a**) virgin PA6, (**b**) PA6-BABA/SA_30_, (**c**) PA6-BABA/SA_50_, and (**d**) PA6-BABA/SA_70_.

The effect of the BABA/SA content on the crystallization behavior of PA6 was elucidated by XRD analysis, as depicted in Figure 3. The 2θ ranges of the diffraction peaks from virgin PA6 and PA6-BABA/SA_30_ are mutually consistent, indicating that they have the same crystalline forms. The XRD curves (Figure 3a,b) include peaks at 2θ ≈ 20.1° and 23.8°, which are typical of virgin PA6, and at 20.2° and 23.0°, which are typical of PA6-BABA/SA_30_, which correspond to the α-crystalline phase. As the BABA/SA content increased from 50 to 70 wt %, a broad peak (2θ ≈ 21.3°) appeared (Figure 3c,d), which indicated that PA6-BABA/SA_70_ comprises mostly the γ-crystalline phase [21]. The transformation from the α to the γ crystalline phase also demonstrated that the crystallizability of PA6-BABA/SA is weakened by the incorporation of long methylene units (BABA/SA) in the PA6 backbone. These data are consistent with the DSC results. 

### 3.4. Thermal Properties of Virgin PA6 and PA6-BABA/SA Copolyamides

**Figure 4:**
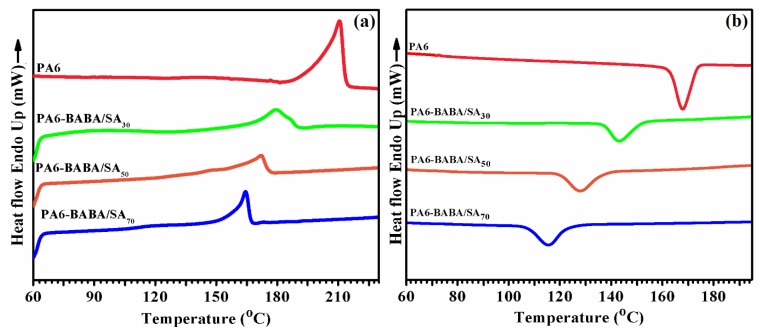
Differential scanning calorimetry (DSC) analyses of virgin PA6 and PA6-BABA/SA copolyamides: (**a**) heating curves (10 °C/min) and (**b**) cooling curves (10 °C/min).

**Figure 5:**
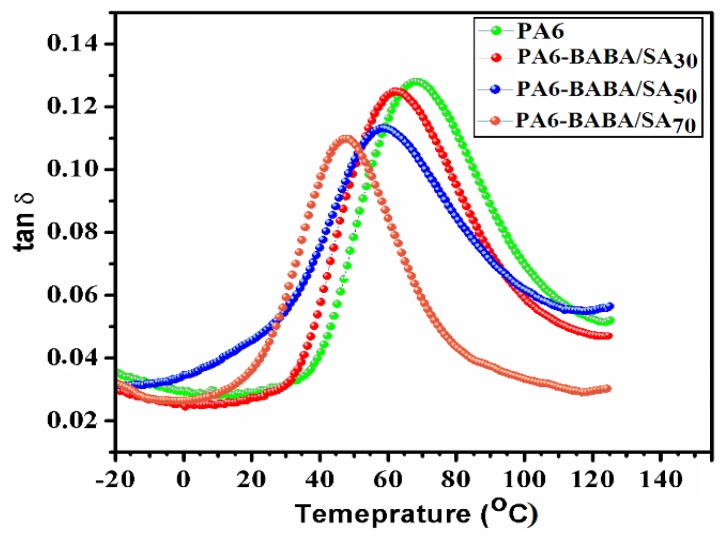
Dynamic mechanical analysis (DMA) analysis of virgin PA6 and PA6-BABA/SA copolyamides.

The effect of the BABA/SA addition on the crystallization and melting behavior of PA6 was assessed using DSC (see Table 2 and Figure 4). The *T*_m_ of the PA6-BABA/SA generally decreased as the number of BABA/SA segments increased, because the BABA/SA disturbed the crystal lattice of PA6, reducing the degree of crystallization and *T*_m_. The weakening of the hydrogen bond and the reduction of the lamellar crystal thickness may cause of this phenomenon [24,25], which was explained by the introduction of long-chain methylene units (BABA/SA) into the PA6 backbone. First, we can notice that the *T*_g_ of copolyamides is difficult to determine from the DSC thermograms because of the lower crystalline phase. Therefore, in this work, the *T*_g_ of all of the copolyamides were determined by using DMA. Comparing the *T*_g_ of neat PA6, an evident reduction in *T*_g_ with the addition of long-chain BABA/SA was observed (see Figure 5 and Table 2). The same behavior was also displayed for *T*_m_ and *T*_c_. Incorporating the long chain length of the repeating unit (BABA/SA) leads to several occurrences that decrease the above-mentioned thermal transition temperature, as follows:

(1) Incorporating a long-chain BABA/SA repeating unit length heightens the mobility of the main PA6 backbone chain. This decreases the activation energy required for glass transition, while also permitting an easier and more effective packing of the polymer chains (reduced *T*_c_), and increasing the entropy change upon melting (reduced *T*_m_). (2) The ratio of methylene:amide linkages also increase with the incorporation of long-chain BABA/SA. This decreases the number of amide linkages per repeat unit, which could possibly partake in hydrogen bonding; these hydrogen bond sites limit chain motion and increase the energy required for the glass–rubber transition (*T*_g_) and melting (*T*_m_). Subsequently, a reduction in the amide linkage density results in reduced *T*_g_ and T_m_ values [26].

### 3.5. Isothermal Crystallization Kinetics

**Figure 6:**
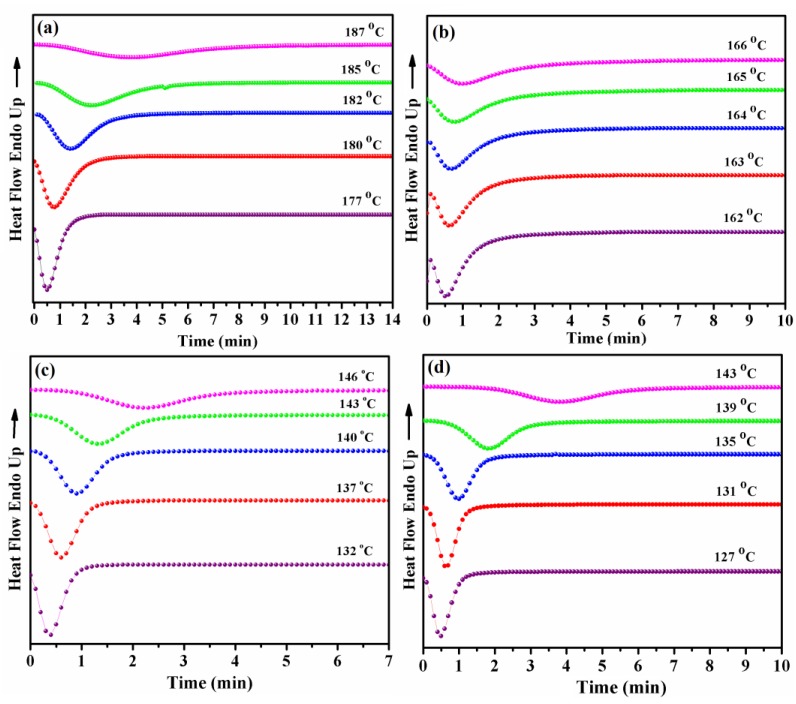
DSC thermograms of isothermal crystallization of (**a**) virgin PA6, (**b**) PA6-BABA/SA_30_, (**c**) PA6-BABA/SA_50_, and (**d**) PA6-BABA/SA_70_ at various *T*_iso_.

To examine in detail the effect of BABA/SA on the crystallization of PA6, the isothermal crystallization kinetics of virgin PA6 and PA6-BABA/SA copolyamides were elucidated by DSC, over a different (178–188 °C, 162–166 °C, 132–146 °C, and 127–143 °C) range of isothermal crystallization temperatures (*T*_iso_). Figure 6 plots the crystallization exotherms of virgin PA6 and PA6-BABA/SA copolyamides at various values of *T*_iso_. The crystallization exothermic curves became broader as *T*_iso_ increased, reflecting a lower crystallization rate, suggesting a nucleation-controlled process [27]. The PA6-BABA/SA samples completed the crystallization more quickly than virgin PA6, at various values of *T*_iso_. 

Table 3 reveals that the crystallization exotherms become more distinct when a small amount of BABA/SA (30 wt %) is incorporated into the PA6, revealing that BABA/SA increases the crystallization rate of PA6. The small quantity of BABA/SA provides nucleation seeds. Consequently, BABA/SA can significantly disturb the crystalline structure of the PA6 and reduce its range of crystallization temperatures. However, as the amount of BABA/SA is continuously increased from 50 to 70 wt % (Figure 6c,d), the range of crystallization times gradually widens and begins to grow into imperfect heterogeneous crystals [28]. Additionally, the incorporation of a large amount of BABA/SA dilutes the crystallizable PA6 component and reduces the crystallization rate. 

To understand better the kinetics of the isothermal crystallization of virgin PA6 and a series of PA6-BABA/SA, the relative crystallinity of polymer X (t) is estimated as a function of time (*t*) at the corresponding *T*_iso_, consistent with Equation (5).

(5) $$X\;\left(t\right)\;=\;\frac{\;X_{c}\;\left(t\right)}{\;X_{c}\;\left(t=\infty \right)}\;=\;\frac{\int_{0}^{t}\frac{\;dH_{c}\;\left(t\right)}{dt}\;dt}{\int_{0}^{t=\infty }\frac{dH_{c}\;\left(t\right)}{dt}\;dt}$$

where $X_{c}\;\left(t\right)$ and $X_{c}\;\left(t=\infty \right)$ are the relative crystallinity at time (*t*) and infinite time, respectively, and $dH_{c}\left(t\right)/dt$ is the heat flow rate. 

**Figure 7:**
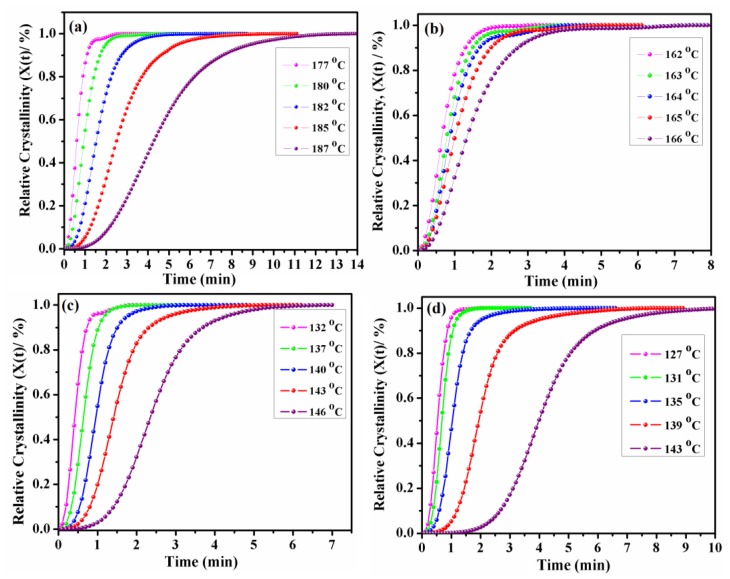
Plots of the relative crystallinity *X*(*t*) versus crystallization time at various isothermal crystallization temperatures (*T*_iso_) for (**a**) virgin PA6, (**b**) PA6-BABA/SA_30_, (**c**) PA6-BABA/SA_50_, and (**d**) PA6-BABA/SA_70_.

Figure 7 plots X (t) for all of the polyamide samples. The isotherm curves of the crystallinity of all of the polyamide samples have a “sigmoid” shape, and shift to the right with an increasing *T*_iso_, showing that the isothermal crystallization rate of virgin PA6 and PA6-BABA/SA firstly increases and then declines with the escalating T_iso_, perhaps because the crystals impinge at a high *T*_iso_. Furthermore, the time required for X (t) to reach its plateau increased with *T*_iso_, reflecting a slower crystallization. Table 3 reveals that the PA6-BABA/SA samples reach the plateau in less time than neat PA6, suggesting that the added BABA/SA may act as a nucleating agent for PA6, and accelerate the overall crystallization process, facilitating its crystallization in PA6-BABA/SA [29].

The isothermal crystallization analysis of the time-dependent relative crystallinity function X (t) is typically performed using the classical Avrami equation, as follows [30,31].

(6) $$X\;\left(t\right)=1-\exp\left(-Kt^{n}\right)$$

where *K* is a crystallization rate constant involving both the nucleation and crystal growth rate parameters, and *n* is a mechanism constant that depends on the form of nucleation and the growth dimension. To obtain the isothermal crystallization parameters, Equation (6) can be further expanded by taking the double logarithm, as follows:

(7)  lg[−ln (1−X(t))]=n lgt+lgK

**Figure 8:**
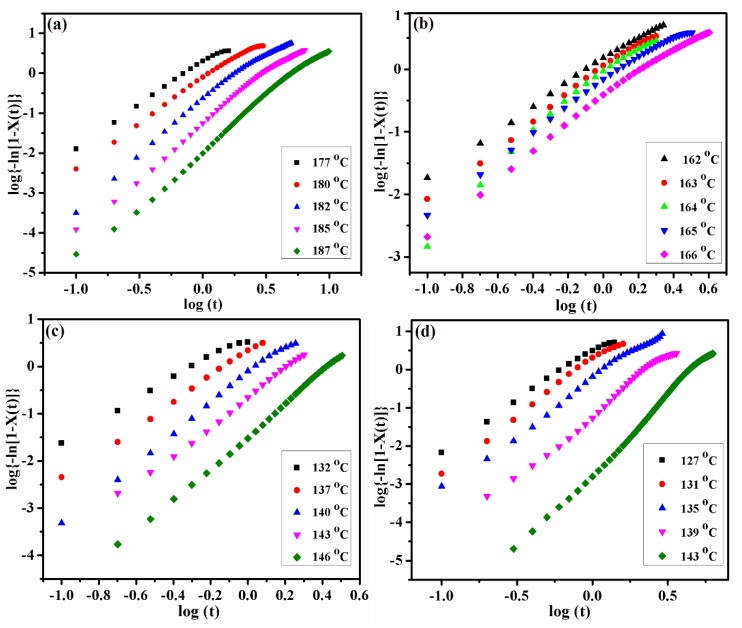
Avrami plots of log{−ln[1−X(t)]} vs. log*t* for isothermal crystallization of virgin (**a**) PA6, (**b**) PA6-BABA/SA_30_, (**c**) PA6-BABA/SA_50_, and (**d**) PA6-BABA/SA_70_ at *T*_iso_.

Figure 8 plots lg[−ln(1−X(t))] as a function of the ln*t* of virgin PA6, and a series of PA6-BABA/SA for various *T*_iso_. A linear regression, y=mx+c, is used to estimate the *n* and *K* values from Figure 8. Most curves in Figure 8 are linear, showing that the Avrami equation describes the isothermal crystallization process of virgin PA6 and PA6-BABA/SA. Accordingly, the values of *K* and *n* at various *T*_iso_ for virgin PA6 and the typical PA6-BABA/SA copolyamides can be calculated, yielding the results in Table 4. 

From Table 4, the values of the Avrami exponent (*n*) clearly increase with the *T*_iso_. The Avrami exponent (*n*) for neat PA6 is 2.09–2.65, which mean that the chains tend to take the 2D growth mechanism. With the addition of BABA/SA_30_, the mechanism of nucleation and the growth of PA6 crystallite are greatly influenced, leading to a decrease of the Avrami exponent (*n*) (1.84–2.44) in the copolyamides. The growth of crystals in the PA6-BABA/SA_30_ copolyamide is probably 1D and 2D, and the nucleation process is heterogeneous and simultaneous under the experimental conditions. After adding 50 and 70 wt % of BABA/SA (*n* = 2.2–3.4 for 50 wt % BABA/SA; *n* = 2.57–3.91 for 70 wt % BABA/SA), the compatibility between PA6 and BABA/SA is increased, which thus promotes the PA6 chains in PA6-BABA/SA_50_ and PA6-BABA/SA_70_ copolyamides to take a growth mechanism of 3D with thermal nucleation.

Table 4 presents the variation of n for the PA6-BABA/SA samples with the BABA/SA content for various *T*_iso_. The value of n decreased as the BABA/SA content increased to 30 wt %, and then began to increase as the BABA/SA wt % content was increased further (to 50 and 70 wt %). The value of n in this study of PA6-BABA/SA_30_ is lower than the values of virgin PA6, PA6-BABA/SA_50_, and PA6-BABA/SA_70_. The smaller n value may be attributable to a faster crystallization mechanism that does not allow for a sufficient time for growth in three dimensions [32,33].

The value of *K* for each polyamide sample decreased with the increasing *T*_iso_, reflecting a decrease in the crystalline growth rate and nucleation rate. The *K* value of PA6-BABA/SA typically exceeds that of virgin PA6 at various *T*_iso_, indicating that the crystallization rate of PA6-BABA/SA exceeds that of virgin PA6. Therefore, *K* increases with the BABA/SA content as a result of the greater flexibility of the BABA/SA units, which may act as a plasticizer, and thus increase the crystallization rate of PA6 in PA6-BABA/SA. This phenomenon occurs because BABA/SA acts (1) as a long-chain or more flexible than PA6 structure, facilitating crystallization, and (2) as a third component that disturbs the regularity of the copolymer molecular chains, retarding crystallization [22]. Qiu et al. obtained a similar result [34].

The crystallization half time (*t*_1/2_) is one of the most interesting and important variables in the study of crystallization kinetics. It is defined as the time required to reach a relative crystallinity of 50%. The *t*_1/2_ can be obtained directly from Figure 7, and can be calculated from Equation (8).

(8) $$t_{1/2}={\left(\frac{\ln2}{K}\right)}^{1/2}$$

Accordingly, the reciprocal of $t_{1/2}$ (i.e., $G=\;\tau_{1/2}=\;1/t_{1/2}$) denotes the crystallization rate. Table 4 presents the values of t_1/2_ for all of the polyamide samples herein. According to the table, for virgin PA6, the $t_{1/2}$ of crystallization increases from 0.63 to 4.66 min, as *T*_iso_ increases from 178 to 188 °C. Similarly, for PA6-BABA/SA_30_, $t_{1/2}$ increases from 0.69 to 1.47 min as *T*_iso_ is increased from 162 to 166 °C, supporting the claim that PA6-BABA/SA crystallizes more rapidly. Figure 6 and Figure 8 verify that the small BABA/SA content acts as a heterogeneous nucleating seed. The $t_{1/2}$ values of PA6-BABA/SA_50_ and PA6-BABA/SA_70_ increase from 0.44 to 2.43 min and 0.57 to 4.43 min, respectively, as *T*_iso_ is increased from 132 to 146 °C and 127 to 143 °C, respectively. This result reveals that the rate of crystallization increases and then decreases as BABA/SA wt % increases.

The increase in the crystallization rate and the fact that the crystallites become imperfect in the PA6-BABA/SA copolymers may be due to the increase in the mobility of the PA6 and BABA/SA chains in PA6-BABA/SA [35]. The variation among the crystallization rates of all PA6-BABA/SA samples can be associated with the size of the phase domains. The mean size of a dispersion phase domain in PA6-BABA/SA_30_ is larger than that in PA6-BABA/SA_50_ or PA6-BABA/SA_70_, so the crystallization rate of PA6-BABA/SA_30_ exceeds those of the other two polymer systems. This fact has already been observed in the literature [36,37]. The $t_{1/2}$ values for all of the PA6-BABA/SA samples are smaller than that of virgin PA6. Therefore, BABA/SA acts as a nucleating agent for PA6 and favors heterogeneous nucleation.

### 3.6. Activation Energy of Isothermal Crystallization

**Figure 9:**
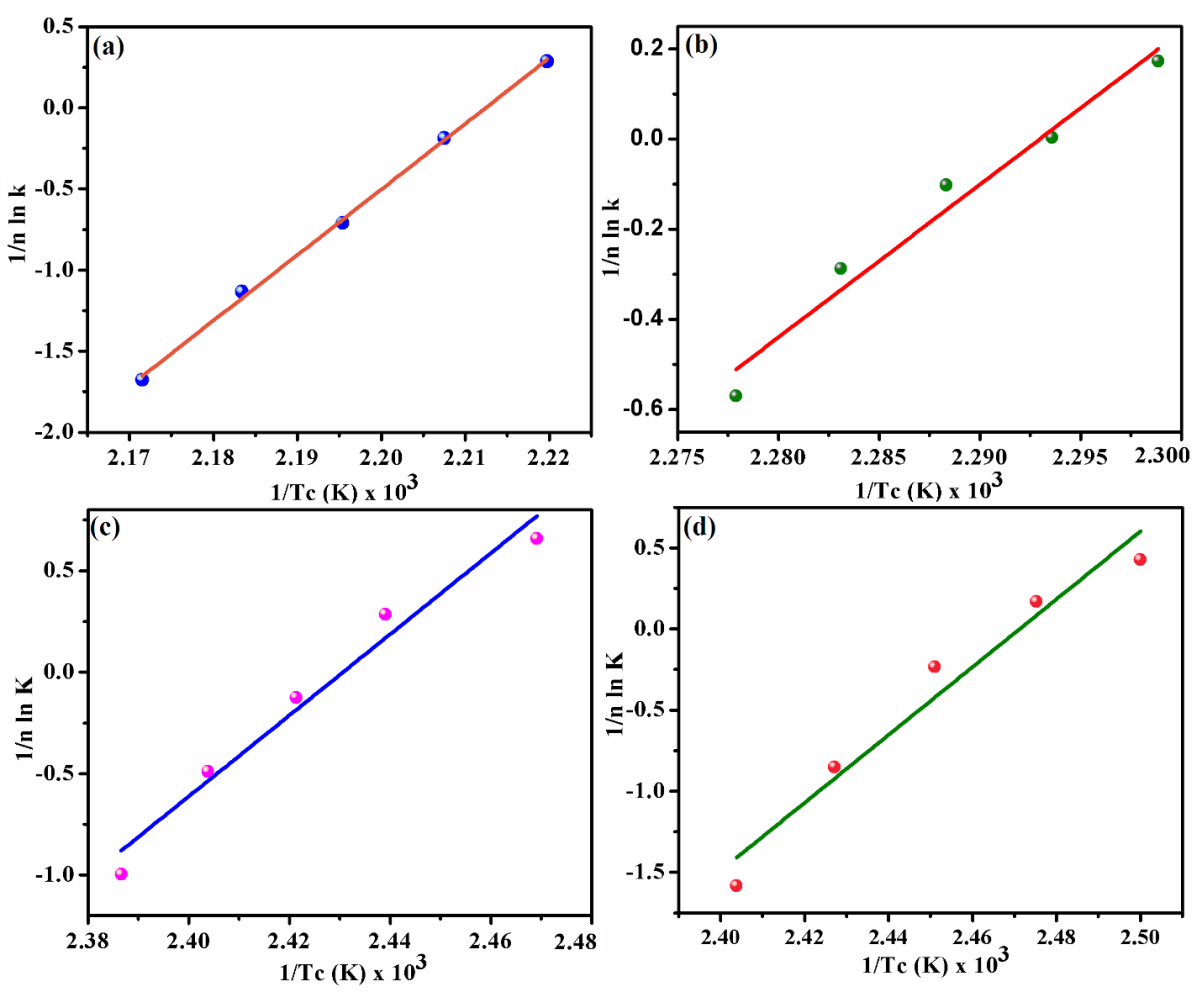
Activation energy for isothermal crystallization of (**a**) virgin PA6, (**b**) PA6-BABA/SA_30_, (**c**) PA6-BABA/SA_50_, and (**d**) PA6-BABA/SA_70_ at various crystallization temperatures.

Based on the assumption that the isothermal crystallization kinetics of virgin PA6 and a series of PA6-BABA/SA are thermally activated and can be used to determine the crystallization energy, the crystallization rate constant parameter (*K*) can be described using an Arrhenius equation, as follows [38]:

(9) $$k^{1/n}={\;k}_{0}\;\exp\;\left(\frac{-E_{a}}{RT_{c}}\right)$$

(10) $$\left(\frac{1}{n}\right)\ln k=\ln k_{0}+\;\left(\frac{-E_{a}}{R}\right)\;\left(\frac{1}{T_{c}}\right)$$

where *k* is the crystallization rate constant, $k_{0}$ is a temperature-independent pre-exponential factor, *n* is the Avrami index, *T*_c_ is the temperature of isothermal crystallization (*K*), R is the ideal gas constant, and $E_{a}$ is the crystallization activation energy (kJ/mol). The slope of a plot of (1/n)lnk against $1/T_{c}$ yields $\Delta E_{a}$ for all of the polyamide samples (Figure 9). The $\Delta E_{a}$ values of the PA6-BABA/SA copolyamides are −281.99, −183.28, and −173.8 kJ/mol, respectively, which are higher than the value of −336.24 kJ/mol for isothermally crystallized PA6. Notably, $\Delta E_{a}$ increases with the wt % of BABA/SA, because the loading of the new comonomer disturbs the regularity and reduces the crystallization order [17]. These results indicate that PA6-BABA/SA with a larger BABA/SA content has lower crystallization $\Delta E_{a}$. As energy must be released as the molten fluid is transformed into the crystalline state, $\Delta E_{a}$ is negative, consistent with the convention.

### 3.7. POM Analysis

**Figure 10:**
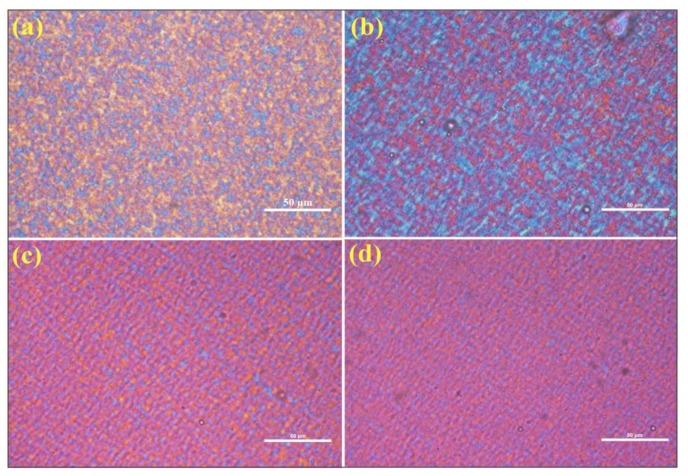
Polarizing microscopy (POM) microscopic images of isothermally crystallized samples of the following: (**a**) virgin PA6, (**b**) PA6-BABA/SA_30_, (**c**) PA6-BABA/SA_50_, and (**d**) PA6-BABA/SA_70_.

The morphology of crystallization was observed by POM, and the photographs of PA6 and PA6 copolyamides are shown in Figure 10. It is found that the radius of PA6 is uniform and the smaller crystals are only several micrometers (Figure 10a). When 30 wt % BABA/SA was added to PA6, the radius of spherulite increased significantly. This increment of the crystal size is more obvious for the PA6-BABA/SA_30_ copolyamide, as shown in Figure 10b. However, after incorporating 50 to 70 wt % BABA/SA, the compatibility between BABA/SA and PA6 is enhanced and the dispersion of BABA/SA in PA6 is improved. Better dispersion of BABA/SA decrease the restriction of the spherulites growth, but, interestingly, some spherulites are found (Figure 10c,d), which grow using the small aggregation of BABA/SA as the nucleus, thus giving strong evidence for the heterogeneous nucleation effect of BABA/SA.

## 4. Conclusions

In this work, PA6 and PA6-BABA/SA copolyamides were synthesized by the melt copolymerization of CPL, which was combined with various wt % of BABA/SA salt. The XRD results reveal that neat PA6 was the α-phase. As the BABA/SA salt content increased above 50 wt %, all of the copolyamides exhibited the γ-phase, indicating that the crystallizability of the copolymer was weakened as the BABA/SA content in the PA6 backbone increased. Similarly, the DSC and DMA results revealed that the *T*_m_ and *T*_g_ of the copolyamides decreased as the BABA/SA content increased. A study of the crystallization kinetics of PA6 and PA6-BABA/SA under isothermal cold conditions using DSC and the Avrami equation demonstrated that the incorporation of 30 wt % BABA/SA into virgin PA6 increased the crystallization rate, which then decreased as the BABA/SA loading was further increased. This result was probably associated with the size of the phase domain, as demonstrated by the half-time of the crystallization. The half-time of the crystallization of the PA-BABA/SA copolyamides was lower than that of neat PA6, revealing that the rate of crystallization increased upon the addition of a BABA/SA segment in the PA6 backbone. The Avrami exponent (*n*) was between two and three, suggesting two-dimensional and three-dimensional growth. The activation energies for isothermal crystallization were determined using the Arrhenius theory, which indicated that PA6-BABA/SA has the higher activation energy.

## Figures and Tables

**Table 1:** Characteristics of virgin PA6 and PA6-BABA/SA prepared by melt polymerization.

Samples	Monomer Feed, wt % Ratio (CPL/BABA/SA) ^a^ wt %	Built-in Composition (CPL/BABA/SA) ^b^ wt %	*RV* dL/g	*M*_w_
PA6	100	-	2.81	20,275
PA6-BABA/SA_30_	70/30	70.5/29.4	2.12	12,652
PA6-BABA/SA_50_	50/50	51.4/48.6	2.32	14,861
PA6-BABA/SA_70_	30/70	30.7/69.3	2.52	17,182

^a^ Determined by weighed-in monomers; ^b^ Determined by ^1^H NMR. CPL—ε-caprolactam.

**Table 2:** Thermal properties and calculated degrees of crystallization of neat PA6 and PA6-BABA/SA copolyamides.

Samples	*T*_m_ (°C)	*T*_c_ (°C)	Δ*H*_m_ (J/g)	*X*_c_%	*T*_g_ (°C)
PA6	211	170	53	28.4	65.9
PA6-BABA/SA_30_	180.4	143.1	45.8	24.5	58.4
PA6-BABA/SA_50_	172.4	128.1	38.4	20.5	52.2
PA6-BABA/SA_70_	164	115.5	28.5	15.3	43.5

**Table 3:** Isothermal crystallization kinetic temperatures (*T*) and crystallization times (min) for neat PA6 and PA6-BABA/SA.

Sample	*T*_iso_ (°C)	Time (min)
PA6	178	0.464
	180	0.796
	183	1.457
	185	2.224
	188	3.701
PA6-BABA/SA_30_	162	0.48
	163	0.601
	164	0.684
	165	0.77
	166	0.974
PA6-BABA/SA_50_	132	0.398
	137	0.59
	140	0.913
	143	1.297
	146	2.314
PA6-BABA/SA_70_	127	0.474
	131	0.663
	135	1.027
	139	1.875
	143	3.87

**Table 4:** Kinetic parameters of isothermal crystallization of virgin PA6 and PA6-BABA/SA copolyamides at various predetermined temperatures.

Sample	*T*_iso_ (°C)	*n*	*K* (min^−*n*^)	*t*_1/2_ (min)	$E_{a}$ (kJ/mol)
PA6	178	2.09	1.82	0.63	−336
	180	2.1	0.68	1.01	
	183	2.4	0.18	1.75	
	185	2.6	0.05	2.7	
	188	2.65	0.01	4.66	
PA6-BABA/SA_30_	162	1.84	1.37	0.69	−282
	163	2.07	1.01	0.84	
	164	2.44	0.78	0.95	
	165	1.93	0.57	1.1	
	166	1.99	1.1	1.47	
PA6-BABA/SA_50_	132	2.22	4.3	0.44	−183
	137	2.71	2.17	0.66	
	140	3.12	0.68	1.01	
	143	3.06	0.22	1.45	
	146	3.4	0.03	2.43	
PA6-BABA/SA_70_	127	2.57	3	0.57	−174
	131	2.84	1.61	0.74	
	135	2.81	0.52	1.11	
	139	3.24	0.06	2.09	
	143	3.91	0	4.43	
